# Exceeding the guidelines: A descriptive study of exercise, pregnancy, maternal and neonatal health outcomes in elite and recreational athletes

**DOI:** 10.1186/s12884-025-07572-6

**Published:** 2025-04-23

**Authors:** Emilie Mass Dalhaug, Birgitte Sanda, Kari Bø, Wendy J. Brown, Jorunn Sundgot-Borgen, Lene Annette Hagen Haakstad

**Affiliations:** 1 Norwegian School of Sports Sciences, Department of Sports Medicine, Oslo, Norway; 2 Akershus University Hospital, Department of Obstetrics and Gynaecology, Lørenskog, Norway; 3 Bond University, Faculty of Health Sciences and Medicine, Gold Coast, Australia, and University of Queensland, School of Human Movement and Nutrition Sciences, Brisbane, Australia; 4 Arendal Gynekologi AS, Arendal, Norway

**Keywords:** Delivery, Obstetric, High-intensity training, Exercise volume, Offspring health

## Abstract

**Background:**

Exercise during pregnancy is associated with numerous health benefits. However, guidelines for elite and recreational athletes, who often exceed general recommendations regarding intensity, duration, and frequency are lacking, and potential risks remain unclear. The aim of the study was to describe exercise levels, pregnancy, and maternal and neonatal health outcomes in elite and recreational athletes.

**Method:**

This study was part of the Strong Mama research project, which was carried out in Oslo, Norway, between October 2022 and February 2024. Sixty athletes (10 elite and 50 recreational) participated in the study. They completed an online survey during late pregnancy and participated in a structured telephone interview six weeks postpartum. The survey and interview collected data on exercise habits, pregnancy experiences, and maternal and neonatal health outcomes.

**Results:**

The athletes maintained high exercise levels during pregnancy, with elite athletes exercising an average of 11.6 h per week (SD 3.2) and recreational athletes exercising 7.0 h per week (SD 2.4). Most athletes resumed exercising within six weeks postpartum. Almost all pregnancies were planned, including six which involved fertility treatment. Most women (76.7%) had spontaneous onset of labor and vaginal deliveries to term (between 36 and 42 weeks). The mean birthweight was 3487 (SD 519.4, range 2600–4775) grams. Two of the elite athletes were diagnosed with gestational diabetes mellitus and two with hypertension during pregnancy. None of the 50 recreational athletes reported any pregnancy complications.

**Conclusion:**

High levels of exercise during pregnancy did not seem to negatively impact maternal or neonatal health in this descriptive sample of Norwegian elite and recreational athletes. However, more research is needed to confirm these findings.

## Background

Regular physical activity during pregnancy is associated with numerous health benefits, including reduced risk of gestational diabetes mellitus, hypertensive disorders, excessive gestational weight gain, fetal macrosomia, anxiety, and prenatal depression [1, 2]. Hence, most national and international guidelines encourage pregnant women to accumulate at least 150 min of moderate-intensity aerobic exercise per week and recommend twice-weekly muscle-strengthening exercises [3–8]. The World Health Organization advises that women who habitually engaged in vigorous-intensity aerobic activity before pregnancy may continue these activities during pregnancy and the postpartum period [3]. However, others recommend that exercise intensity should be below 80% of heart rate reserve [6] or 8 on a scale of 1–10 [8]. Few guidelines include advice on higher levels of activity, high-intensity exercise, and heavy load resistance training [4], which are common in elite and recreational athletes [9–11].

In recent years, many athletes have challenged the societal narrative that they should “slow down” during pregnancy. Exceeding the current activity recommendations is assumed to carry potential risks because the safety of high-intensity, long-duration, or high volumes of exercise during pregnancy is unclear [12]. Several studies have shown that sport and exercise participation decline during pregnancy in elite and recreational athletes [13], as well as in the general population [14–16]. This may reflect concerns among pregnant women and healthcare providers that high levels of exercise during pregnancy may increase the risk of pregnancy and delivery complications [17, 18].

A recent systematic review and meta-analysis of pregnancy outcomes in elite athletes found no elevated risk of having babies with low (< 2500 g) or high (> 4000 g) birthweight, preterm birth (< 37 weeks), miscarriage, cesarean section, instrumental delivery, episiotomy, or prolonged labor and perinatal tears, compared with the general pregnant population [12]. However, the authors rated the quality of evidence as “low” or “very low” for all outcomes, mostly because of the low number of studies.

In 2017, an International Olympic Committee (IOC) expert committee highlighted the need for more research into maternal and neonatal outcomes in elite athletes, including fertility problems, medical conditions, pregnancy complications, delivery, and exercise during pregnancy and postpartum, and how these compare with recreational athletes [19]. Thus, the aim of the current study was to describe exercise levels, pregnancy, and maternal and neonatal health outcomes in elite and recreational athletes who exceed the physical activity recommendations, especially in relation to high-intensity exercise and heavy-load resistance training.

## Method

This observational study was part of the Strong Mama research project. The primary aim of the experimental part of the project was to investigate the fetal and maternal physiological responses to high-intensity interval training and heavy load resistance training in elite and recreational athletes. Pregnant elite athletes are a unique population, and there are very few athletes on a high level getting pregnant annually. Thus, our recruitment target for the Strong Mama research project was 60 participants, including as many elite athletes as possible. This sample size is substantially larger than most studies on this topic [20–23].

In the current paper, we report the findings from a survey that was completed at about 30 weeks of gestation and from structured interviews at approximately six weeks postpartum. All data were collected in Oslo, Norway, between October 2022 and February 2024.

### Participants

Participants were pregnant elite and recreational athletes between gestation weeks 26 and 35, with a singleton pregnancy and with the ability to understand verbal and written Norwegian or English. Following the consensus set by the IOC expert committee [9], we define elite athletes as individuals who are part of any national team or other high-level representative teams in any sport, as organized by a National Sports Federation. This includes participants in elite leagues for team sports such as handball and football. It is important to note that all the elite athletes did high-volume exercise (≥ 240 min/week) during their pregnancies. Following this, recreational athletes were defined as women who reported regular exercise both for fitness and competition ≥ 240 min/week, including sessions of high-intensity exercise and/or heavy load resistance training, but who were not members of any high-level representative team.

There were no age or parity-related inclusion criteria, but women were excluded if they experienced any medical or obstetric contraindications to exercise during baseline selection [5].

Elite athletes were recruited in collaboration with the Norwegian Olympic Sports Center as well as national team doctors and support teams at the various sports federations. Recreational athletes were recruited through social media, newspaper articles, healthcare clinics in Oslo, and word of mouth. Two hundred and twelve women (12 elite and 200 recreational athletes) initially expressed interest in participation and were contacted by the research coordinator. Twenty-eight were excluded as they did not meet the 240 min/week exercise inclusion criterion at 26–35 weeks of gestation. Of the remaining 184 women, the first 60 who met the criteria and were available to participate were included in the study.

## Assessment methods and procedures

### Questionnaire during pregnancy

Participants completed an online survey at 26–35 weeks gestation. The survey was based on a prior study by Sundgot-Borgen and colleagues [24] and included questions about demographic characteristics, exercise during pregnancy, pregnancy history, pregnancy symptoms, and indicators of health and well-being, including eating disorders and life satisfaction (see Table 1).

**Table 1:** Dimensions assessed, and main variables recorded to answer the research questions

Dimensions assessed	Main variables
Demographic characteristics	Age, marital status, education
Exercise in pregnancy for recreational athletes	Number of years exercising more than 3 times/week Type of exercise (running, strength training, ball sports, CrossFit, Yoga/Pilates, dancing, cycling, aerobics, ski sport, combat sports, swimming, orienteering and other, please specify) Frequency of exercise (hours/week) during pregnancy (in total and separately for endurance training at low, moderate, and high intensity, and strength training with low, moderate, and high load).
Exercise in pregnancy for elite athletes	Number of years participating in organized sport Type of sport (handball, football, volleyball, racket sport, orienteering, athletics, swimming, powerlifting, X-country skiing, biathlon skiing, alpine skiing, snowboard and freestyle, gymnastics, cycling, triathlon, combat sports, hockey, dance, golf, basketball, sailing, skating, paddling, ski jumping and other, please specify). Frequency of exercise (hours/week) in the first, second, and third trimesters of pregnancy (in total and divided into endurance training at low (intensity sone 1–2), moderate (intensity sone 3–4), and high intensity (intensity sone 5–8), strength training with low, moderate, and high load, technique training, mental training, and pelvic floor muscle training).
Pregnancy history	Pregnancy week, number of pregnancies, number of children, time to conceive, and use of fertility treatment.
Pregnancy symptoms	Participants were asked to rate how often they experienced the following pregnancy complaints (16 in total) on a 4-point scale (0 = never, 1 = rarely, 2 = sometimes, 3 = often), and rate how much these complaints limited their daily activities (not limited at all, limited a little or limited a lot) [25]: nausea/vomiting, fatigue, poor-quality sleep, low back pain, pelvic girdle pain, Braxton Hicks contractions in everyday life, Braxton Hicks contractions during exercise, constipation, mood swings, birth anxiety, feeling depressed, edema, dizziness, shortness of breath, hemorrhoids and headache.
Health	Height and pre-pregnancy weight. These variables were used to calculate pre-pregnancy BMI.
Eating disorder	Assessed using the Norwegian version of The Eating Disorder Examination (EDE-Q [26]), with a cut-off set to 2.5 indicating symptoms of eating disorders [27].
Satisfaction with life	Assessed using the Satisfaction with Life scale [28]. The following classifications were used: total score 5–9 = extremely dissatisfied, 10–14 = dissatisfied, 15–19 = slightly dissatisfied, 20 = neutral, 21–25 = slightly satisfied, 26–30 = satisfied, 31–35 = extremely satisfied [28].
Rating of health	Participants were asked to rate their current health on a scale ranging from 1 (poor) to 5 (excellent).

### Structured telephone interview postpartum

Approximately six weeks after their expected due date, participants were contacted to schedule a telephone interview. This structured interview was administered by trained research assistants and included questions about the athlete’s birth experience and birth outcomes. Participants were asked to report from their hospital records on weeks of gestation at birth, type of birth (including induction of labor and indication for induction), use and type of analgesia, use of augmentation, instrumental delivery (vacuum, forceps, cesarean delivery (acute and elective)), episiotomy, grade of perineal tears, postpartum bleeding, and perceived exertion and labor pain on a scale from 1 to 10, with 10 being the worst possible pain. Also, neonatal outcomes, such as birth weight and length, head circumference, Apgar score, and admission to NICU, and maternal outcomes, such as gestational weight gain, gestational hypertension, pre-eclampsia, and gestational diabetes mellitus were recorded. Participants were also asked about their exercise habits during the last stage of pregnancy and following birth, pregnancy-related complaints, and quality of life.

### Statistical analyses

Descriptive statistics were conducted using Statistical Package of Social Sciences (SPSS) v. 28 (IBM, Armonk, New York, USA). Due to the small sample size, it was not feasible to conduct any tests for statistical significance. Therefore, results are presented descriptively without statistical inferences. Descriptive data are presented as frequencies with percentages or means with standard deviation and ranges, as appropriate. Pre-pregnancy BMI and gestational weight gain were used to calculate the number of women who gained weight below, within, or above the Institute of Medicine (IOM) recommendations [29]. Responses of ‘sometimes’ and ‘always’ were used to indicate the occurrence of complaints.

## Results

### Participant characteristics

Out of 60 participants, 59 (10 elite and 49 recreational athletes) completed the questionnaire at an average gestational week of 30.1 (SD 2.9, range 26–36 weeks). All 60 participants (10 elite and 50 recreational) completed the postpartum telephone interview at an average of 7.1 weeks (SD 1.8, range 4–12 weeks). Among the elite athletes, five were endurance athletes, four were ball sport athletes, and one competed in CrossFit. All competed at the highest level nationally or internationally in their sport before pregnancy and were planning to return after giving birth. In the recreational athlete group, the majority participated in strength training (*n* = 41), running (*n* = 39), cycling (*n* = 20), CrossFit (*n* = 13), and X-country skiing (*n* = 12). Some were sub-elite ultrarunners, triathletes, X-country skiers, CrossFit athletes, powerlifters, or former elite athletes, while others participated in high volumes of exercise for enjoyment, health benefits, and to facilitate a faster return to pre-pregnancy form after labor.

Participants’ demographic characteristics are shown in Table 2. Almost all were partnered and had higher education. For the majority of participants, this was their first pregnancy.

**Table 2:** Characteristics of the elite and recreational athletes from the pregnancy questionnaire. Results are presented as frequencies with percentages and means with standard deviations (SD)

	Elite athletes *(n = 10)*	Recreational athletes *(n = 49)*
	**Mean (SD)**	**Mean (SD)**
Age (years)	31.1 (3.10)	32.0 (3.47)
Pre-pregnancy BMI (kg/m^2^)	22.7 (2.81)	22.4 (1.97)
Parity	0.1 (0.31)	0.4 (0.60)
	**n (%)**	**n (%)**
Pre-pregnancy BMI category Underweight (< 18.5) Normal weight (18.5–24.9) Overweight (≥ 25.0)	0 (0.0) 7 (70.0) 3 (30.0)	0 (0.0) 41 (83.7) 8 (16.3)
Higher education (University/College)	10 (100.0)	46 (93.9)
Married or partnered	10 (100.0)	48 (98.0)
Primiparous Multiparous	9 (90.0) 1 (10.0)	33 (67.3) 16 (32.7)

### Exercise during pregnancy

On average the recreational athletes had exercised regularly (≥ 3 times/week) for 16.2 (SD 6.6) years pre-pregnancy. The elite athletes had participated in organized sports for 23.0 (SD 4.2) years. On average, the elite and recreational athletes exercised for 11.6 (SD 3.2, range: 7–17) and 7.0 (SD 2.4, range: 4–17) h/week during pregnancy, respectively. The elite athletes did more endurance exercise (7.4 h/week; comprising 4.5 (SD 3.6) h/week at low intensity, 2.0 (SD 1.1) h/week at moderate intensity, and 0.9 (SD 1.5) h/week at high intensity) than the recreational athletes (4.8 h/week; comprising 2.4 (SD 2.3) h/week at low intensity, 1.5 (SD 1.0) h/week at moderate intensity and 0.9 (SD 0.8) h/week at high intensity).

Amounts of resistance training were similar in the two groups (elite: 3.0 h/week, comprising 0.6 (SD 0.8) h/week with high load, 1.5 (SD 0.6) h/week with moderate load and 0.9 (SD 0.9) h/week with low load; recreational: 3.0 h/week, comprising 0.9 (SD 1.0) h/week with high load, 1.3 (SD 1.0) h/week with moderate load and 0.8 (SD 1.0) h/week with low load).

### Exercise in the postpartum period

All the elite (*n* = 10) and most of the recreational (*n* = 45) athletes had started exercising within six weeks postpartum, with some initiating exercise within a few days after giving birth. All athletes reported a slow progression of exercise. The most commonly reported exercises within the first six weeks were pelvic floor muscle training, moderate-intensity stroller walks, low-load strength training, and running. Elite athletes reported exercising on average 9.9 (SD 4.0, range: 4–18) h/week, and the recreational athletes 5.5 (SD 3.2, range: 1.5–21) h/week at the time of the telephone interview. After six weeks, the athletes reported doing strength training (*n* = 40), running (*n* = 32), pelvic floor muscle training (*n* = 17), cycling (*n* = 9), Crossfit (*n* = 3), and X-country skiing/roller skiing (*n* = 3). One elite and five recreational athletes reported that they exercised at the same level as before pregnancy. However, it should be noted that four of the recreational athletes were between nine and eleven weeks postpartum at the time of the interview.

### Maternal health

#### Pregnancy planning and fertility

Five recreational athletes (10.2%) and one elite athlete (10.0%) had experienced a miscarriage before this pregnancy. For most elite and recreational athletes, the pregnancy was planned (*n* = 8 (80%) and *n* = 40 (81.6%), respectively) and spontaneous (*n* = 9 (90%) and *n* = 44 (89.8%), respectively). Among those requiring fertility treatment, in vitro fertilization (IVF) (*n* = 4) and ovulation induction (*n* = 2) were the most common treatments.

#### Pregnancy complaints and complications

In both groups, the most frequently reported pregnancy complaints were fatigue, poor-quality sleep, Braxton Hicks contractions in everyday life and during exercise, mood swings, and low back pain (Table 3). Two elite athletes were diagnosed with gestational diabetes mellitus during pregnancy, and another two elite athletes were diagnosed with hypertension. None of the 50 recreational athletes reported any pregnancy complications.

**Table 3:** Pregnancy complaints in elite and recreational athletes from the pregnancy questionnaire. Results are presented as frequencies with percentages

	Elite athletes *(n = 10)* *n* (%)	Recreational athletes *(n = 49)* *n* (%)
Fatigue	8 (80.0)	40 (81.6)
Poor-quality sleep	8 (80.0)	34 (69.4)
Braxton Hicks contractions in everyday life	4 (40.0)	23 (46.9)
Braxton Hicks contractions during exercise	5 (50.0)	22 (44.9)
Mood swings	5 (50.0)	22 (44.9)
Constipation	2 (20.0)	21 (42.9)
Low back pain	5 (50.0)	19 (38.8)
Shortness of breath	2 (20.0)	12 (24.5)
Pelvic girdle pain	5 (50.0)	10 (20.4)
Nausea/vomiting	4 (40.0)	9 (18.4)
Headache	1 (10.0)	9 (18.4)
Edema	2 (20.0)	8 (16.3)
Birth Anxiety	4 (40.0)	5 (10.2)
Feeling depressed	4 (40.0)	5 (10.2)
Dizziness	2 (20.0)	5 (10.2)
Hemorrhoids	3 (30.0)	5 (10.2)

#### Gestational weight gain

The average gestational weight gain was 14.3 kg (SD 4.6, range: 8–22) in the elite athlete group and 13.1 kg (SD 3.4, range: 4–20) in the recreational athlete group. Based on the IOM weight gain recommendations [29], three (30%) elite athletes and fifteen (30%) recreational athletes gained below the recommended levels, while three (30%) elite and nine (18%) recreational athletes gained more than recommended. Weight gain in four (40%) elite and 26 (52%) recreational athletes was within the guidelines.

#### Eating disorders and satisfaction with life

One recreational athlete and none of the elite athletes had a Global EDE-Q score indicating symptoms of eating disorders during pregnancy.

Most elite (*n* = 9) and recreational (*n* = 36) athletes were either satisfied or extremely satisfied with their lives during pregnancy. 60% (*n* = 6) of elite athletes and nearly 88% (*n* = 43) of recreational athletes rated their health as ‘very good’ or ‘excellent’ during pregnancy. The corresponding numbers postpartum were 70% (*n* = 7) and 88% (*n* = 44) for elite and recreational athletes, respectively.

### Delivery outcomes

The average gestational week for birth was 39.9 (SD 1.4, range 36 + 0–42 + 0). One recreational athlete gave birth prematurely in gestational week 36 + 0, and one elite and one recreational athlete gave birth post-term in gestational week 42 + 0.

The majority of women had spontaneous onset of birth with vaginal (non-instrumental) deliveries, and the epidural was the most used analgesia during labor (Table 4). On average, elite and recreational athletes rated the pain experienced during labor as 9.5 (SD 0.7) and 9.3 (SD 1.1), respectively, on a scale from 1 to 10. Further, they rated labor exertion using the same scale as 8.9 (SD 1.2) and 7.8 (SD 2.2), respectively.

**Table 4:** Birth outcomes in elite and recreational athletes from the postpartum telephone interview. Results are presented as frequencies with percentages

	Elite athletes *(n = 10)* *n* (%)	Recreational athletes *(n = 50)* *n* (%)
Preterm birth (< 37 weeks of gestation)	0 (0.0)	1 (2.0)
Vaginal delivery Spontaneous onset Induced onset	9 (90.0) 6 (60.0) 3 (30.0)	48 (96.0) 40 (80.0) 8 (16.0)
Cesarean section		
Emergency Elective	1 (10.0) 0 (0.0)	2 (4.0) 0 (0.0)
Instrumental delivery		
Vacuum Forceps	1 (10.0) 0 (0.0)	7 (14.0) 0 (0.0)
Use of analgesia		
Epidural Nitrous oxide	7 (70.0) 2 (20.0)	23 (46.0) 3 (6.0)
Episiotomies	3 (30.0)	10 (20.0)
Degree of perineal tears		
None to second degree Third to fourth degree	10 (100.0) 0 (0.0)	50 (100.0) 0 (0.0)
Postpartum bleeding		
>500 ml >1000 ml	1 (10.0) 1 (10.0)	8 (16.0) 4 (8.0)
NICU		
< 48 h ≥ 48 h	2 (20.0) 0 (0.0)	2 (4.0) 1 (2.0)

### Birthweight and neonatal health

The mean birth weight of babies born to elite athletes was 3407 (SD 290.4) grams, and to recreational athletes it was 3503 (SD 554.7) grams. No athletes had babies with a birth weight < 2500 g, while ten recreational athletes (20%) had babies with a birth weight > 4000 g, of whom four (8%) had a birth weight > 4500 g. The mean Apgar score was 9.0 (SD 0.0) and 8.7 (SD 0.8) after one minute and 9.3 (SD 1.6) and 9.7 (SD 0.7) after five minutes for elite and recreational athletes, respectively. The babies of three recreational athletes had a low Apgar score (< 7) after one minute, and one of the elite athletes had a baby with a low Apgar score after one and five minutes. Eight athletes had missing values for the Apgar score.

## Discussion

The literature has raised concerns regarding the potential adverse effects of high-volume maternal exercise on neonatal health, labor, and birth outcomes [10]. Thus, it is interesting to note that in this descriptive study, we had relatively few cases of miscarriages, fertility problems, low birth weights, pre-term births, and cesarean deliveries, and few athletes having symptoms of an eating disorder. This suggests that in our small group of highly fit participants who exceeded the exercise recommendations during pregnancy, high exercise volumes did not seem to pose as significant a risk to maternal and neonatal health as previously thought. However, more research is necessary to confirm this suggestion.

### Exercise during pregnancy and postpartum

Research in the general pregnant population indicates that physical activity levels generally decrease as pregnancy progresses [13–15, 30], with only about 10% of women in a Norwegian cohort continuing to exercise at least four times per week in late gestation [15]. The elite and recreational athletes in our study had significantly higher exercise levels during pregnancy. This finding aligns with previous research, which showed that women with high levels of physical activity before conception tend to maintain higher levels of physical activity during pregnancy than those who are more sedentary [30, 31]. Although women are often recommended to have a slow progression of exercise the first six weeks after giving birth [3, 32], most elite and recreational athletes in our study began exercising at weeks 0–6 postpartum. This is consistent with a recent systematic review and meta-analysis [33]. However, a study by Sundgot-Borgen and colleagues [24], involving Norwegian elite athletes and active controls (regularly physically active for a minimum of 150 min per week), found that only 32% of controls returned to their sport and exercise routine within the first six weeks postpartum. The earlier return to exercise observed in our recreational athletes compared to the active controls in Sundgot-Borgen and colleagues’ study [24] may be due to differences in inclusion criteria and that we included a highly active group of recreational athletes. Thus, our participants were probably more active than the active control group and possibly more eager to resume exercise postpartum, which might explain the difference.

### Maternal health outcomes

According to the Medical Birth Registry of Norway, 5.2% of children born in 2021 were conceived through assisted fertilization, of which 2.6% were through IVF [34]. In our sample, 10% of babies were conceived through fertility treatment. This aligns with Thornton and colleagues’ [35] study finding that 8% of former elite athletes required fertility treatment to become pregnant. In contrast, Sundgot-Borgen and colleagues’ [24] survey of 68 Norwegian elite athletes and active controls found that only two required fertility treatment, both of whom were in same-sex relationships. Given the small size of our sample (60 participants), it is important to emphasize that we cannot draw conclusions about these fertility rates in comparison to the general population. Thus, more research is warranted.

A recent systematic review and meta-analysis found that the prevalence of symptoms of eating disorders in pregnant women was 4% [36]. This is in line with prevalence data from the Norwegian Mother and Child Cohort Study (MoBa), which reported that one in 21 women had symptoms of eating disorders during pregnancy [37]. Further, a Swedish study found that 13% of postpartum mothers were suffering from an eating disorder [38]. Given that eating disorders are more prevalent among elite athletes than the general population [39], it is noteworthy that only one recreational athlete in our study had Global EDE-Q score indicating symptoms of an eating disorder during pregnancy. However, it has been suggested that pregnancy itself does not necessarily trigger or increase the risk of persistent eating disorders for elite athletes [24], contrary to what has been observed in non-athletic women [40]. This finding underscores the complexity of these disorders and the multifactorial influences involved, highlighting the need for further research.

Despite their high exercise levels, two elite athletes were diagnosed with gestational diabetes mellitus during pregnancy and another two with gestational hypertension, while none of the recreational athletes were diagnosed with either conditions. This contrasts with the findings of Thornton and colleagues [35], who reported a significantly lower incidence of gestational diabetes in their sample of retired elite athletes than in the general population. Among the elite athletes with hypertension, both gained weight within the guidelines, whereas those diagnosed with gestational diabetes exceeded the recommendations. While excess weight gain may have contributed to the gestational diabetes, it is important to note that ten other athletes who also exceeded the weight gain recommendations did not develop the condition. This suggests that other factors, such as genetic predisposition, dietary habits, or the specific physiological demands of their sports, may play a role. Additionally, we lack detailed information on the timing, severity, or management of these conditions, which limits our ability to fully assess their impact. Given the small sample size, these findings may also have occurred by chance and should be interpreted with caution. Further research is needed to better understand the prevalence and underlying factors of these conditions in elite athletes during pregnancy.

### Delivery outcomes

In our study, we observed a trend towards higher analgesia rates among elite athletes compared with recreational athletes. It has been hypothesized that female elite athletes may have hypertrophied pelvic floor muscles that do not sufficiently adapt or stretch during vaginal delivery [41, 42]. This could potentially lead to higher rates of instrumental delivery, prolonged labor, failure to progress in labor resulting in emergency cesarean sections, and severe perineal tears (third- to fourth-degree) [41–43]. However, this hypothesis has been refuted with systematic reviews concluding that neither general exercise [43–45] nor specific pelvic floor muscle training [46] during pregnancy has shown a negative effect on vaginal delivery. This implies that the small trend toward higher analgesia rates among elite athletes warrants further investigation and may be influenced by factors beyond the physical characteristics of the athletes.

Although we found a slightly higher percentage of vacuum deliveries amongst our sample of elite and recreational athletes compared with data from the general population (13.3% vs. 8.6%) [34], the percentage of emergency cesarean deliveries (5.0% vs. 6.9%) was similar, and none in our sample had third- to fourth-degree perineal tears. Thus, our results on cesarean sections support the previous literature which suggests that participating in high volumes of exercise during pregnancy does not increase the risk of emergency cesarean sections [12, 13].

Excessive gestational weight gain has been identified as a risk factor for macrosomia [47], and thus instrumental deliveries [48]. Compared with data from the Norwegian Birth Registry [34] we found a higher percentage of babies born with macrosomia (> 4500 g) (6.6% vs. 2.7%) and a higher number of vacuum deliveries. However, out of eight vacuum deliveries, only one baby weighed > 4500 g. The mother of this baby had a normal pre-pregnancy BMI and gained below the IOM weight gain recommendations [29], suggesting other factors may have contributed to the slightly higher number of vacuum deliveries. In our study, most women who underwent a vacuum delivery reported prolonged labor as the reason for this, with three of these cases also noting that the baby was in the occiput posterior position.

### Neonatal health outcomes

A major concern for pregnant athletes is the potential impact that training and competition might have on neonatal health and development [49]. High-intensity exercise is associated with a redistribution of blood flow, and therefore nutrients, away from the fetus towards skeletal muscle [10, 20, 50]. Thus, a theoretical concern is that regular high-intensity exercise may impair fetal growth and development. Compared with data from the Medical Birth Registry of Norway [34], results from our sample were similar for mean birth weight (3487 g in our sample vs. 3485 g in the general population), mean gestational age at birth (39.9 (SD 1.4) weeks vs. 39.2 (SD 1.9) weeks) and percentage of post-term deliveries (3.3% vs. 3.4%). Further, we found a lower percentage of preterm births (1.6% vs. 5.8%), and none in our sample gave birth to a baby with low birth weight (< 2500 g) (4.7% in the general population). Our results on birth weight concur with systematic reviews and meta-analyses of data from non-elite [51] and elite athletes [12], which have reported that physical activity does not negatively impact birth weight or the risk of low or high birth weight. Data from the general population show that 17% of babies born in Norway have a birth weight > 4000 g and 2.7% >4500 g [34]. Interestingly we found a slightly higher percentage of babies with high birth weight than in the general population, and none of the athletes gave birth to a baby with low birth weight. This is in contrast to the proposed inverted U-shape relationship between physical activity and birth weight, with low-to-moderate exercise volume in pregnancy associated with increased birth weight, while high-intensity exercise and a high exercise volume are associated with a decreased birth weight [50]. Still, the slightly higher number of babies with high birth weights in our sample might be explained by Clapp’s hypothesis that in response to the transient reduction in oxygen and nutrient availability during exercise, blood flow is enhanced at rest [50]. Aligning with our results, exercise during pregnancy does not appear to increase the risk of preterm birth in athletes [12, 24, 52] or non-athletes [53, 54] and might even have a slight protective effect against preterm birth [3, 52, 53]. Thus, it may be assumed that participating in high exercise volumes during pregnancy does not increase the risk of birth weight extremes or preterm birth in elite and recreational athletes.

### Methodological considerations

While we appreciate the efforts of McKay and colleagues [55] to establish a standardized framework for characterizing participants based on their training and performance, we encountered difficulties in categorizing our elite and recreational athletes within this system. In our study, the performance levels among elite athletes varied widely, ranging from those who have won Olympic and/or world medals (Tier 5: World Class), to those competing at an international level (Tier 4: Elite/International), and those competing in elite national leagues (Tier 3: Highly Trained/National). Equally, our recreational athletes, who regularly exercised more than three times per week, would be placed in Tier 2 (Trained/Developmental), according to McKay’s system. However, many of these athletes did not identify with a specific sport or train with the intention to compete, characteristics more aligned with Tier 1 (Recreationally Active). This tier represents individuals who meet the World Health Organization’s activity guidelines, encompassing 35–42% of the global population. Our reluctance to categorize our recreational athletes in the Tier 1 group is due to their exercise volume, which ranged between 4 and 17 h per week, with the majority exceeding 300 min per week. This level of activity appears to surpass the typical expectations for the recreationally active category. As such, we believe that further refinement of this classification system may be necessary to accurately represent the wide range of activity levels within the “recreational athlete” population.

The main strength of the current study is that it is one of the first worldwide to investigate factors associated with pregnancy in elite and recreational athletes who exceed current exercise recommendations. The study employed a comprehensive methodological approach, collecting data both through an online survey during late pregnancy and via a structured telephone interview postpartum. This allowed for a thorough exploration of exercise habits, pregnancy experiences, and maternal and neonatal health outcomes. Also, we specifically targeted elite and recreational athletes, a group often overlooked in pregnancy and exercise research. This focus allowed for a more nuanced understanding of the impact of high-volume, high-intensity exercise during pregnancy. Also, our study not only examined exercise during pregnancy but also included the postpartum period, offering a more complete picture of the athletes’ experiences.

The main limitation of this study is the small sample size which precludes statistical comparison of prevalence estimates in the two groups of athletes and with population estimates, especially for outcomes that are relatively rare. The primary aim of the Strong Mama study was to investigate the acute effects of high-intensity interval training and heavy load resistance training on fetal well-being, and the sample size was larger than in most prior studies on this topic [20–23]. Given the low number of athletes who become pregnant each year, recruiting them for studies can be challenging. Therefore, we aimed to include as many elite athletes as possible within the project’s timeframe. As researchers have highlighted the need for more data on birth and offspring outcomes in elite athletes [56], we chose to present data for elite and recreational athletes separately to inform future research on exercise levels, pregnancy, and maternal and neonatal health outcomes in elite athletes.

The addition of a less active control group could have been valuable but was not feasible for this study. Thus, we chose to compare our results with data from the Medical Birth Registry of Norway [34]. Not surprisingly, our participants’ exercise levels differed from those of the general pregnant population [30], and the athletes had a lower mean pre-pregnancy BMI than Norwegian pregnant women in general [34]. It is worth noting that three elite athletes and eight recreational athletes in our study were classified as overweight based on BMI. As BMI does not differentiate between the different body components, an individual with a high proportion of fat-free mass relative to height might have a high BMI value without being overweight. This is corroborated by Torstveit and Sundgot-Borgen [57], who found that BMI is not a valid measure for assessing body composition in female elite athletes and should be used with caution in non-athletes. Thus, it is unlikely that the BMI status of these women impacts their overall health. In addition, as we only included women without medical or obstetrical contraindications, our sample might have been healthier than the regular pregnant population, and our results may not be applicable or generalizable to all pregnant individuals.

Data on exercise, pregnancy, and maternal and neonatal outcomes are self-reported and therefore subject to social-desirability bias, over and underestimation of exercise, and misclassification of exercise intensity [58, 59]. However, objective measures of physical activity (e.g. accelerometry) were not feasible for the current study. On the other hand, most of our participants tracked their exercise using apps like Strava, making the reporting more reliable.

## Conclusion

Findings from this small exploratory sample of Norwegian elite and recreational athletes suggest that engaging in high volumes of exercise, including high-intensity exercise and heavy load resistance training, during pregnancy does not appear to negatively impact maternal or neonatal health outcomes. However, it is important to note that these results may not be generalizable to all pregnant women, especially those without a history of regular, high-intensity, high-volume exercise.

## Data Availability

The data that support the findings of this study are available from the corresponding author (EMD), upon reasonable request.

## Abbreviations

BMI: Body mass index
EDE-Q: Eating Disorder Examination Questionnaire
IOC: International Olympic Committee
IOM: Institute of medicine
IVF: In vitro fertilization
MoBa: The Norwegian Mother and Child cohort study
NICU: Neonatal intensive care unit
SD: Standard deviation
SPSS: Statistical Package of Social Sciences
